# 144. Antifungal Use Trends in Hospitalized Adults in the United States, 2016-2020

**DOI:** 10.1093/ofid/ofab466.346

**Published:** 2021-12-04

**Authors:** Emily A Kaip, Ernie Shippey, Conan MacDougall

**Affiliations:** 1 University of California, San Francisco Medical Center, San Francisco, California; 2 Vizient Inc., Dallas, Texas; 3 University of California San Francisco School of Pharmacy, San Francisco, California

## Abstract

**Background:**

Surveillance of antimicrobial use is a cornerstone of antimicrobial stewardship, though antifungal (AF) use is less frequently characterized. AFs are a major driver of inpatient costs and their use both reflects and drives changes in fungal susceptibility patterns. We report on trends in AF use in a large sample of United States hospitals over time including predictors of AF use.

**Methods:**

We performed a retrospective analysis of adult inpatient visits between 2016 and 2020 at hospitals contributing data to the Vizient Clinical Database/Clinical Resource Manager (www.vizientinc.com). Inpatient use of systemically administered AFs was investigated as a function of study quarter, diagnosis code, and underlying immunosuppressive condition. Changes in AF use were modeled using logistic and negative binomial regression.

**Results:**

We examined over 23 million admissions across 470 hospitals, 43% of which were classified as teaching hospitals and 54% of which performed solid organ transplants. During the study period, 4.03% (951,284/23,565,493) of admissions were billed for one or more of the study AFs. Among admissions receiving AFs, 86% received an azole, with the most frequently used agent being fluconazole, which accounted for for 46% of total AF days. Likelihood of AF receipt during admission increased by quarter (OR 1.012, p< 0.001), controlling for length of stay, presence of fungal infection, hematologic malignancy (HM), or solid organ transplant (SOT). Odds of any receipt and days of therapy (DOT) of fluconazole, isavuconazole, posaconazole, and echinocandins increased over the study period while those of voriconazole, itraconazole, and flucytosine decreased; odds of receipt of amphotericin products increased while DOT decreased; flucytosine receipt odds increased while DOT did not change. Only 30% of admissions with AF use were associated with a documented fungal infection, with 93% of these episodes documented as candidiasis. Admissions associated with SOT or HM represented 2% and 3% of all patient-days, but 11% and 25% of total AF days, respectively.

Antifungal Utilization

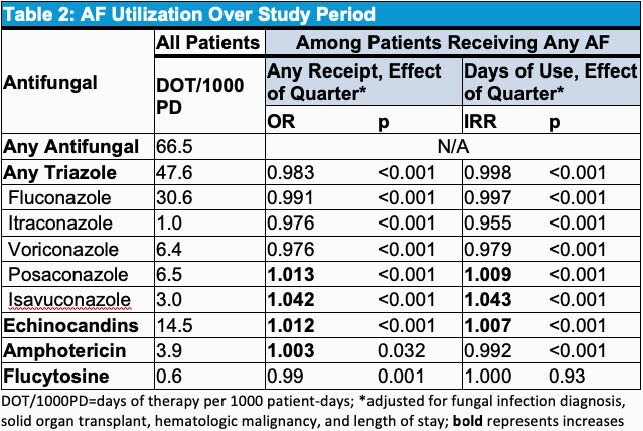

**Conclusion:**

AF use increased significantly over the study period, with changes across agents and classes. Most AF use occurred in the absence of administratively documented infection and was more common among SOT and HM patients.

**Disclosures:**

**All Authors**: No reported disclosures

